# On Fracture of the Basio Cranii

**Published:** 1879-09

**Authors:** 


### Article V.

### On Fracture of the Basio Cranii. Read to McLean County Medical Society.

Several years ago, G. L, a tinner by trade, aged 24 years, of temperate habits, healthy, strong constitution, short figure, fell through a hatchway in the third story of a building on the square, down into the cellar, a distance of about 36 feet and was carried home almost dead. The accident happened in November, about 8 o'clock in the morning, and half an hour afterward I was called to see him, and found him in deep sopor; no external irritants would produce any reflex action of the nerves or nerve center; pulse was full and slow, 50 per minute; respiration superficial and slow, 16 per minute; eyelids were closed, pupils dilated, not reacting to light; conjunctiva could be touched, but patient did not flinch. No ribs nor bones of extremities were broken. On the head I found a wound 1½ inches long, extending from linea aronata superior of left side, over protuberantia occipitalis, to linea aronata of the right, longer on the right than on the left side. Upon entering with the finger, the surface of the occipital bone was found smooth; no cracks could be felt. From the right ear blood ran out drop by drop, but not squirting. On offering him water, patient was able to swallow a little. He lay entirely motionless; occasionally he would take a deeper inspiration, followed by deeper expiration and moaning. When I visited him in the evening, he was almost in the same condition as in the morning; the only noticeable change was, that his pulse had become a little fuller and quicker, 58 per minute, and the body was warmer. Had passed water without knowing it; bowels had not acted. The second day till evening passed without change; he was apathic to everything and did not notice anything; still he swallowed liquid food, water and medicine when it was given; blood still oozed out of the ear, but it was somewhat serous. Discharges from bowels, and urine, came away unconsciously. About 8 o'clock in the evening pulse and temperature rose, and he commenced to scream, like you hear it from insane persons, and he continued to cry in the same way for two consecutive days, with but little interruption.

On the fourth day the brain became a little more active; he could hear that questions were put to him, but the steady answer was, "I want to go out." He could not change his position; he retained the one which was given to him, and he only moved his arms occasionally. From the ear no blood ran out on the third day, but a pretty copious serous discharge, tinged with blood, had taken its place. This discharge became perfectly clear on the fourth day, and continued for fourteen days, in such quantity as to keep his pillow wet. Afterward it became more pus like, diminished in quantity, and slowly disappeared. On the fifth day, after having rested better the previous night, having had a three hours sleep, he showed some improvement. He was more quiet; on being asked if he knew me he said, "Yes, you are the doctor." But he could not remember my name. In fact, his memory was very weak, and he could not recollect the names of the most common things. He hunted, for example, with his arms for something, and when he was asked what he wanted, he said, "I want my legs," instead of "my pants." He could distinguish the difference between light and darkness, but could not see anything, and this blindness lasted till the tenth day after the accident. His recovery was very slow and gradual, his mental faculties, intellect, volition and memory were like those of an idiot; he would ask the same questions over and over, apparently without his understanding what was answered. Bodily he grew stronger, his appetite became good, he slept well, bowels were regular, he could move his arms easily, but not his legs, and when he was put upon these, they sank in powerless. I already feared that he would remain an idiot and cripple all his lifetime, when, four weeks after the accident had happened, the first ray of light fell into the darkness of his mind. Then he remembered a certain sum of money, dollars and odd cents, which he had left in his vest pocket the very morning he received the injury. From thence he continued to improve mentally and bodily, and four weeks later he was able to be up and walk a few steps, but he put his feet wide apart, to have a broader basis for the support of his body. As soon as the feet were put close together he lost the equilibrium. In the right ear hearing was imperfect; still, with the time it became good. His mental faculties became good, and after the lapse of a year, besides a dizziness when he was working on a roof, or in the sun, no sign of his dangerous sickness could be seen. The following year, the great sensibility of the brain to the heat of the sun, and the vertigo, had disappeared, and ever since he has enjoyed perfect health.

I had to deal with a case of fracture of the base of the skull, and made the diagnosis by the copious discharge of blood, followed by the long continued and copious discharge of serum, from the ear. Either alone would not warrant us to make diagnosis of fracture at the base, because there are other lesions attended by a flow of blood or serum from the ear. By keeping the following points in view, we will not be liable to make a mistake:

*First.* The source of blood might be a ruptured membrana tympani, or the injured mucosa lining the cavitas tympani. Both membranes are well supplied with blood vessels, but these are of small caliber, and the quantity of blood discharged from them cannot be copious, nor last long. Soon a clot will be formed, and on removing it the ruptured tympanum can be plainly seen. When we make the paracentesis membranæ tympani, only a few drops of blood escape, and this gives us the proof that the bleeding from ruptured membrane must be insignificant.

*Second.* More copious bleeding occurs when the anterior wall of the meatus audit. externa is broken, what happens sometimes when the condyles of the maxilla inferior are driven into it by a force striking the chin. Copious bleeding takes place, too, when the cartilaginous portion of the meatus is severed from the osseous. But in both cases the external injuries will guide us to find the proper source of bleeding.

When the base is fractured then the blood comes either from the ruptured blood-vessels of the pars petrosa, or from the vessels of the brain and dura mater. The vessels which can be injured are numerous and important, near the posterior wall of the cavitas tympani passes the sinus transversus; its lower wall is separated from the ingularis interna only by a thin lamella of bone. Over the roof goes the meningea media, and just at the upper part of tuba eustachus, lies the carotis interna. The arteries are seldom ruptured, because they are more elastic than the veins and coherent with the bones. But the veins being firmly attached to the sulcus, in which they lie, and having less elasticity, are more frequently injured, and the bleeding must be in case of fracture at the base, copious, sometimes very profuse.

If the serous discharge would be always liquor cerebro-spinalis, then its presence alone would be sufficient to prove a fracture at the base. But this is not the case. There are, it is true, very few instances recorded from trustworthy authorities, where after injury to the head a serous discharge from the ear lasted for several days, and still, when the patients died either from the injury or some time afterwards from other causes, not the least sign of ruptured dura or fractured bone could be found. The explanation of this phenomenon is difficult and no satisfactory answer has been given yet. Some thought it was produced by a hypersecretion of the mucosa in consequence of inflammation, as a case is on record, where, within 24 hours after a cold, so much liquid had gathered in the cavity of the tympanum, that the paracentesis of the membrana had to be made, a large quantity of serum escaped and the flow continued for 8 days. Others said it was perilympha. Be it what it may, such cases are extremly rare, and in the greatest majority of cases the serum oozing out from the ear after injury to the head, is liquor cerebro-spinalis, and I mentioned the above examples only to show that for making a sure diagnosis of fracture at the base, we need the two moments, copious discharge of blood from the ear followed by a long-continued and copious serous discharge. Of course the absence of these symptoms does not exclude the possibility of fracture at the base, because the membrana tympani is not always torn, and in such cases blood and serum would find their way out through mouth and nose, being discharged through eustachian tube.

Fractures at the base may be either direct or indirect, that is to say, the bone may either break at the place where the force hits or at a distance from it. Direct fractures are of rare occurence in civil practice, as the base is well protected in front by the facial bones, and at the back by the strong ligaments and thick muscles of the neck. There are in fact only two places from whence it can easily be attacked, the orbital and nasal cavities. Indirect fractures happen most frequently. The mechanism of such fractures has been a mystery for a long time and many hypotheses have been brought forward to explain it. Some thought that they were produced by irradiation, others said that coup and countercoup was the cause of them. None of these explanations covered the ground. Bruns was the first to give a satisfactory explanation by the elasticity of the skull. In order to show this elasticity he put the skull between the arms of a vise, measured its diameters with a compass, applied pressure and measured again. Then he found that while he had shortened one diameter the others had been lengthened, and on taking off pressure, all diameters had returned to their former size. If the pressure was continued beyond certain limits the bone would invariably break. But these limits were not the same in all skulls. He could compress the skull of a grown person three-fifths of an inch without breaking it, while that of a boy 12 years old broke when the diameter was shortened only one-fifth of an inch. As soon as pressure ceased the fractured bones returned to their former position. So he proved the elasticity of the skull; he proved that by the force hitting the skull the diameter is shortened in the direction of the force, while the others are lengthened; and that when the force is greater than the power of expansion of the bone then the cohesion of the particles will cease—the bone will break. This happens at a place where is the least resistance, but depends, too, on the direction and intensity of the force and the form and density of the instrument by which force is applied. The base, being considerably flat and composed of several bones differing in thickness and elasticity, with angles, cavities, channels, foramina is more liable to these indirect fractures than the other bones of the skull, which are more rounded, homogen in composition and more elastic. Besides this, the spinal column supporting the base and being firmly attached to a part of it, resists any considerable extension of the diameter downwards, and so the bones next to it break the easiest. For this reason we find generally a fracture of the os petrosum, which by its position and its brittleness is the most exposed. The fracture is almost always in the form of fissure, which commences at the porus acoust. intern. passes through labyrinth and tympanum and reaches the meatus auditor. externus. The course of the fissure gives us the reason why blood and liquor cerebrospinalis runs out from the ear. The dura lines the porus acust. intern. and the arachnoidea surrounds the nerv. facial. and acousticus and accompanies them into the meatus internus itself; both membranes must be torn, when the bone breaks and a communication is established with the sub-arachnoidal space. The liquid gets into the cavity of the tympanum either through the fissure or through the ruptured fenestra ovalis, and from thence through the ruptured membrana tympani outside. Union takes place by ossification, the same way as on other bones, only it takes longer time and the union is not so firm for several causes.

1. The voluntary or involuntary action of the muscles produces at the ends of the fractured bones a slight friction, which is the proper stimulus for an accelerated flow of blood to marrow bone and periosteum (ubi irritatio, ibi affluxus) and consquently for plastic exudation and production of callus. No such friction takes place when the bones at the base are broken. The fragments remain in the same position, firm and rigid, in which they are left after the accident has happened.

2. The tela ossea is less active because the dura is separated from the bone by the fracture and its nutrient vessels are torn. Therefore,

3. The production of callus is left almost exclusively to the tela medullaris of diploe.

The fracture as such does not need any treatment. We do not need to coapt the ends of fragments, they remain as they are left after the injury; the bleeding stops spontaneously. If it is so profuse that we are certain of the rupture of a large vessel, then patient will die soon. Some surgeons have tried trepannation in such cases and put a ligature round the meningea media ; but the operation was not very successful and the patients died.

Our whole attention must be directed to the lesions which the brain invariably must suffer, to commotion, contusion and compression. When after a fall or stroke on the head the injured person becomes insensible, falls down with pale face, closed eyelids, superficial respiration, slow, small, filiform pulse, we speak of a commotion of the brain. After a while patient may take a few deep inspirations, awake, open the eyelids and get up ; when trying to walk he will stagger, complains of pains in the head, feels dizzy and weak. Some time elapses yet before he will recover perfectly and resume his work. These we call light cases. In severe cases he will not move, he remains in deep coma, nothing will rouse him, no external irritants will produce the slightest reaction. Pupils may be dilated or not, but light has no influence on them, extremities are cold, pulse slow and weak. Patient may either die soon during coma, or after a few hours perhaps even days, reaction takes place, pulse beats quicker, temperature rises and even symptoms of congestion of the brain may appear. Ultimately patient may recover or other spmptoms appear which tend to show that other lesions of the brain had taken place. The deeper the coma and the longer it lasts, the less we are authorized to make the diagnosis of simple commotion. What is commotion ? It was said that the vibrations of the skull were communicated to the substance of the brain, that oscillations took place and that in consequence of these a change was produced in the molecular arrangement of the brain. Such explanation is probably wrong ; because it is not possible that after such a serious injury to the substance of the brain, as the derangement of molecules must be, the normal function of the brain could be re-established in so short a time as we see it in light cases.

Experiments, too, have proved that no such oscillation takes place within the brain. A glass balloon is filled with gelatine of the consistency of the brain, and then threads of dark colored silk are disseminated through it. Now if on striking the glass, waves would pass through the substance, a slight quivering of the threads could be seen. But not even with a good microscope can such movement be observed; and as the only result of the stroke, we see that the whole substance moves, when enough room is left in the balloon for the gelatine to move. Others opened the head by a trephine, inserted a needle into the brain, and attached a thin piece of paper to the head of the needle. On striking with a hammer, the brain protruded through the opening, but the paper on the needle did not move. Others filled the skull with sand; and all came to the conclusion that no oscillations take place, and that only the whole brain moves, that it moves in the direction of the stroke, that it accommodates itself to the changed diameter of the skull, and that the consequence of such accommodation is a contusion, either at the place where the force hits, or at the opposite side. Autopsies made soon after death from commotion, generally show signs of contusion, destruction of brain substance, or capillary apoplexies; and several eminent surgeons were by these post mortem examinations led to deny the existence of commotion, and believed that death was produced either by contusion, or that, when no signs of contusion could be found, some lesion in other organs had taken place, which had escaped the attention of the examiner. But there is not any doubt that most careful examinations have been made, where no anatomical lesion could be found, or where the signs of contusion were slight and in comparatively unimportant portions of the brain, that the question arose: What did the patient die of? Certainly not of the slight contusion; less so, as we know that by rifle balls sometimes considerable portions of the brain are destroyed without lethal effect. On looking at the symptoms, we see that intellect, action of the heart and respiration, motility and sensibility of the patient is altered, that all functions of the brain are disturbed. Such a general disturbance of the functions can only be produced by a disturbance of the circulation of the blood and what follows it, and incompetent nutrition. When after being struck with a hammer the head of an animal is opened, the brain looks pale and does not move, and only when the animal rallies the vessels fill up again. The same phenomenon can be seen in the eye, when the pupils are well dilated by atropine; the vessels of retina are plainly visible, as soon as you strike the animal on the head, they become either invisible or can only faintly be distinguished, till the animal has rallied. We see, therefore, that when the head is struck an anæmia of the brain takes place followed very likely by a venous stasis and certainly by an improper nutrition of the brain. This explains in the most simple manner the functional derangements, which characterize the symptoms of commotion, and gives us the reason why slight cases recover so soon, but why still some time has to pass before they rally. We are still at a loss to know the exact cause of this trouble in the circulation; very likely it is produced by a reflex paralysis of the vaso-motor nerves. The tonus of the walls gets lost, arteries do not contract, insufficient supply of oxygen stops function of brain, vagus makes heart action slow and weak, blood goes into the veins but slow, and as they lose their tonus a stasis is produced. According to the above explanations, we are, therefore, led to the belief that commotio cerebri belongs to the symptoms of shock, and that the paralysis either exists in the blood-vessels of the brain alone, or a general paralysis of the vessels ensues.

In our case during the first two days the most prominent symptoms were those of commotion, loss of intellect and sensibility; inability to move, weak and slow action of the heart. After this the circulation through the brain was partially re-established, which was shown by the increased temperature and quicker movement of the heart. I say partially established, because there was still absence of intellect and vision and the legs remained paralyzed, showing that still an impediment existed for the proper nutrition of the brain, and this was caused by a clot of extravasated blood, or, to use the technic term, by compression. I am afraid I have imposed upon your time and patience already too long, and abstain from going into a detailed discussion of compression. I content myself to say, that it represents an impediment to circulation and nutrition, and that when compression is sudden and the substance, producing pressure, large, then the clinical symptoms are almost identical with those of commotion, and when compression is so great as to stop nutrition, death will always occur. If compression comes on gradually, then the brain will accommodate itself to the defect, nutrition and only symptoms of local pressure will appear. In our case the extravasation of blood took place very likely immediately after the fall, but pressure did not get to a high pitch because the fracture served as a safety-valve through which blood and afterwards liquor cerebro-spinalis could escape, and when commotion seemed to have passed, only symptoms of local pressure, loss of intellect and vision and paralysis of the legs remained. With the resorption of the clot, the brain resumed gradually its normal function. I said the fracture acted as a safety-valve, but it is the road, too, through which the bacteria enter the interior of the skull and produce inflammation and suppuration of the meningea.

Our therapeutical action must be directed to the following points:

1. We must counteract the shock and avoid everything, that depresses the heart's action. We put, therefore, patient to bed, cover him well with blankets, put warming pans to his feet and hot cloths to abdomen. If patient can swallow, we give him stimulants, with caution.

2. We must try to give tonus to the paralyzed vessels, and avoid, when reaction takes place, congestion. Ice bags to the head are the best remedy. As adjuvants, two remedies may be given, which are known to contract the vessels, bromide of potash and ergot.

3. We must promote resorption. For this purpose we stimulate the organs of secretion and excretion, salivary glands, skin, kidneys and intestines.

The following is the course of treatment I pursued: During the first day I kept the body warm, applied ice bags to the head, and gave occasionally whisky with water. Towards evening when pulse became a little quicker, I stopped the whisky and gave bromide of potash and calomel with jalap, alternately every two hours till I had produced ptyalism and profuse evacuations. Then I quit giving calomel, continued with jalap alone, but not so often as before and combined the bromide with acetate of potash, giving it every four hours. On the fourth evening, when there was more activity of the brain, I gave 10 grains of Dover's powder to allay the restlessness. The next morning I had patient sponged off with tepid water, and from thence up to the time of his recovery he received two spongings daily. The temperature of the water was diminished gradually till it was ice cold; after being sponged patient was well rubbed. Ice bags, bromide and acetate of potash were used till the function of the brain was normal. Dover's powder and jalap were given when necessary. Diet was regulated according to his necessities. To avoid, if possible, septic poisoning, the ear was washed out every day with weak solution of carbolic acid (1.100) and clothes wet with the solution kept on it, and changed as often as soaked with serum. The legs improved under the use of the cold water spongings and liniments, which I had applied three times daily. Very likely it was the massage that helped them most. When patient was able to be about, I forbade him strictly to read, and enjoined him to keep away from everything that could excite him. For two months more he took the elixir of pyrophosph. of iron and bark as a tonic.

---

DIFFERENTIATION OF CANCER OF STOMACH FROM DYSPEPSIA BY THE ABDOMINAL TEMPERATURE. (*La France Médicale, July* 9, 1879).

At the Societé Clinique, Paris, communicated some interesting facts relative to local morbid temperatures in certain affections of the abdomen. A patient presented marked symptoms of dyspepsia, with progressive wasting away. The general appearance favored the supposition of cancer of the stomach, but there was no vomiting, no tumor in the epigastric region. The epigastric temperature was carefully taken, and the average (we suppose) was 37.3°, while the temperature in the axilla did not exceed 36.8°.

The patient died and an autopsy revealed cancer of the posterior wall of the stomach, extending to the large curve. The orifices were not invaded.

The normal temperature of the epigastric region is given as 35.5°, and the statement made that in simple dyspepsia elevation of temperature does not occur.

